# Healthcare Professionals Perspectives on Telemedicine for Patients With Chronic Diseases: A Qualitative Study

**DOI:** 10.1111/nhs.70157

**Published:** 2025-06-04

**Authors:** Natassia Kamilla Juul, Mette Juel Rothmann, Anne Dichmann Sorknæs, Katrine Schultz Overgaard, Søren Auscher, Kenneth Egstrup

**Affiliations:** ^1^ Research Unit for Internal Medicine and Emergency Department Odense University Hospital Svendborg Denmark; ^2^ Department of Clinical Research University of Southern Denmark Odense Denmark; ^3^ Center for Innovative Medical Technology – CIMT Odense University Hospital Odense Denmark; ^4^ Steno Diabetes Center Odense Odense University Hospital Odense Denmark; ^5^ OPEN, Open Patient Data Explorative Network Odense University Hospital, Region of Southern Denmark Odense Denmark

**Keywords:** COPD, healthcare professionals, heart failure, home‐monitoring, qualitative, telemedicine, video consultation

## Abstract

**Aim:**

To explore healthcare professionals' perspectives on telemedicine, specifically video consultations and digital home monitoring, in specialized care for chronic heart failure and chronic obstructive pulmonary disease. The study focused on opportunities and barriers in hospital and primary care.

**Methods:**

A qualitative study with semi‐structured interviews within a phenomenological‐hermeneutic framework. The study was conducted in Denmark, where 12 participants (eight nurses, four physicians) were interviewed between May and September 2022. Half held leadership roles. Nine worked in hospitals, and three in primary care. Data were analyzed using Braun and Clarke's thematic analysis, adhering to Consolidated Criteria for Reporting Qualitative Research guidelines.

**Results:**

Three key themes were generated through interpretive analysis: (1) balancing challenges and benefits of virtual care, (2) strengthening cross‐sectoral collaboration, and (3) empowering patients through telemedicine. Video consultations were well received but required user confidence. Digital home monitoring faced skepticism, particularly regarding patient‐measured data, but improved self‐care and decision‐making. Cultural gaps hindered cross‐sectoral collaboration.

**Conclusion:**

Unfamiliarity with telemedicine and cross‐sectoral gaps is a key barrier, highlighting the need for training and mutual understanding. Addressing these issues is crucial for successful implementation and improved patient care.

**Summary**

- This paper examines attitudes and experiences of healthcare professionals with telemedicine in outpatient clinics, focusing on the care and treatment of patients with chronic widespread diseases.
- It identifies the benefits and challenges of video consultations and home monitoring from the perspective of HCPs in both hospital and primary care settings.
- It highlights key areas that must be addressed to support the effective implementation of telemedicine solutions in clinical practice.

## 1 Introduction

This paper presents experiences with the use of telemedicine solutions in outpatient clinics from the perspective of healthcare professionals (HCPs). The clinics are specialized in the care and treatment of individuals suffering from the two common diseases, chronic heart failure (CHF) and chronic obstructive pulmonary disease (COPD). This study offers insight into the current benefits and challenges associated with the adoption and implementation of video consultations and home monitoring into outpatient care. These insights apply to clinical practice in both hospital and primary care settings, as navigating through a digital transformation forces new skills and places new demands on cross‐sectoral collaboration. While the study is situated in the Danish healthcare system, the findings contribute to the growing global discourse on digital transformation in chronic care (Masterson Creber et al. 2023).

### 1.1 Background

The prevalence of the two public health diseases, CHF and COPD, is rising globally. Both are chronic diseases that significantly affect quality of life, causing physical and psychological challenges. Health economic trends show a shift toward outpatient and virtual care, with fewer hospital beds and limited resources (McDonagh et al. 2021; WHO 2024). This has led to an increasing demand for digital healthcare solutions.

Recent advances in medical treatment have alleviated symptoms and extended life expectancy, but research has shown that empowering patients through education is equally important (McDonagh et al. 2021). Teaching patients to recognize and manage early symptoms of deterioration not only enhances self‐care but also improves their overall quality of life (Cichosz et al. 2020; Pedersen et al. 2017; Aamodt, Lycholip, et al. 2020). Telemedicine, including home monitoring tools and virtual communication platforms, can support this educational process by actively involving patients in disease management (Cichosz et al. 2020; Pedersen et al. 2017; Vestergaard et al. 2020; Ware et al. 2020; Aamodt, Lycholip, et al. 2020). Home monitoring enables patients to track symptoms and key health metrics, thereby encouraging more proactive engagement in their care. Home monitoring devices, which allow patients to measure blood pressure, oxygen saturation, or weight, can increase patient awareness of symptom changes and support early intervention. Video consultations further enable regular follow‐ups and ensure that patients receive timely advice and support. However, the successful use of such tools depends on patient digital literacy. Older adults may face challenges due to limited skills, access, or confidence, which are factors that should be considered when designing digital healthcare solutions to support self‐care (Masterson Creber et al. 2023). Studies show that both home monitoring and video consultations facilitate early detection of deteriorations. This allows HCPs to intervene promptly, prevent hospital readmissions, and reduce healthcare costs (Ware et al. 2020; Aamodt, Lycholip, et al. 2020). This proactive approach benefits both patients and healthcare systems by preventing disease progression and reducing disruptions to daily life.

The healthcare sector is experiencing major changes driven by digitalization, which incorporates video consultations as an important and growing part of healthcare services. In Denmark, the political recommendations from the Health Reform 2024 state that patients in outpatient clinics should be offered virtual consultations, if appropriate (Ministry of the Interior and Health's Communications Unit 2024). This applies to situations where no physical examination is needed, and the patient feels confident with a virtual consultation. The goal is to improve accessibility, reduce logistical challenges, and optimize healthcare resources while maintaining quality care (Ministry of the Interior and Health's Communications Unit 2024). Shifting to telemedicine requires HCPs to develop new skills in virtual communication and respond to home monitoring data. Virtual communication places distinct demands on HCPs, including the ability to actively listen, interpret facial expressions, and recognize non‐verbal cues during video consultations (Ford and Reuber 2024; Nguyen et al. 2024; Randhawa et al. 2019). These skills are essential for establishing trust, building relationships, and fostering mutual understanding between HCPs and patients. They are particularly important in managing chronic diseases, where effective communication forms the foundation for patient‐centered care (Nguyen et al. 2024).

Despite increasing international interest in telemedicine and political recommendations for its use, a gap exists in understanding the perspectives of HCPs on implementing telemedicine solutions in specialized chronic care. Some HCPs have embraced virtual consultations, while others show skepticism related to the implementation (Rosenstrøm et al. 2024).

In the present study, we aimed to explore HCPs perspectives on telemedicine, including the use of video consultations and digital home monitoring, in the specialized care and treatment of patients with CHF and COPD. The focus was specifically on the opportunities and barriers associated with integrating these technologies in both hospital and primary care settings. Although the study was conducted in Denmark, the findings reflect broader issues relevant to healthcare systems globally.

## 2 Materials and Methods

### 2.1 Study Design

The study employed a qualitative design, using semi‐structured interviews within a phenomenological‐hermeneutic framework (Green et al. 2013). This approach aimed to gain in‐depth insight into HCPs experiences with the use of telemedicine solutions in specialized patient care and treatment. The study was based on a contextualist understanding of knowledge and viewed experience as subjective and shaped by context. This alignment reflected the interpretative focus of reflexive thematic analysis and the phenomenological‐hermeneutic framework (Braun and Clarke 2022). The Consolidated Criteria for Reporting Qualitative Research (COREQ) was used as a checklist to ensure comprehensiveness and transparency in reporting the study (Tong et al. 2007).

### 2.2 Setting

Participants were recruited from two outpatient clinics at a university hospital in Denmark and from a semi‐rural municipality with approximately 50 000 residents, comprising both small towns and agricultural areas. The outpatient clinics included a heart failure clinic and a pulmonary disease clinic. Additionally, we conducted an interview with one physician that represented the general practitioners.

### 2.3 Participants and Recruitment

The HCPs were recruited using purposive sampling (Green et al. 2013), and were invited to participate voluntarily either by email or face‐to‐face. Participants were eligible if they were nurses, physicians, or healthcare leaders from hospital or municipal settings with clinical or organizational experience in telemedicine for patients with CHF or COPD. The sampling aimed to ensure variation in profession (nurses, physicians), role (clinical staff, leaders), and sector (hospital, municipality). Purposive sampling included HCPs with experience‐based knowledge of telemedicine. As use of telemedicine remains limited in some settings, particularly primary care and among hospital physicians, participants were selected for their familiarity with video consultations and/or home monitoring. This supported rich, relevant data generation. Several leaders also had dual roles, providing both clinical and managerial practice.

### 2.4 Data Collection

Interviews were conducted (May 2022 to September 2022) by a female clinical nurse specialist (MScN) with prior qualitative research experience and professional interest in telemedicine. She was a colleague of three participants, with no personal relationship to the others. The interviews were carried out either face‐to‐face in a private room at the hospital or virtually through telephone or a videoconference system.

Individual, in‐depth semi‐structured interviews were conducted, as this method produces rich and detailed accounts (Green et al. 2013). The interview guide was developed in collaboration with a concurrent study (ADLIFE) conducted in the same clinical department (Moll et al. 2023). It was guided by the Health Organization Technology‐fit (HOT‐Fit) Framework (Yusof et al. 2008) and inspired by existing literature (Pedersen et al. 2017; Randhawa et al. 2019). The questions relevant to this study are included in Appendix. We conducted 12 interviews with HCPs specialized in CHF or COPD. The sample size was considered sufficient to capture variation across settings and professions, guided by the concept of information power (Malterud et al. 2016). All interviews were recorded and transcribed verbatim.

### 2.5 Data Analysis

Data analysis was guided by Braun and Clarke's thematic analysis framework (Braun and Clarke 2022), following six structured phases: (1) data familiarization; (2) systematic data coding; (3) generating initial themes; (4) developing and reviewing themes; (5) refining, defining, and naming themes; and (6) writing the report.

**FIGURE 1:**
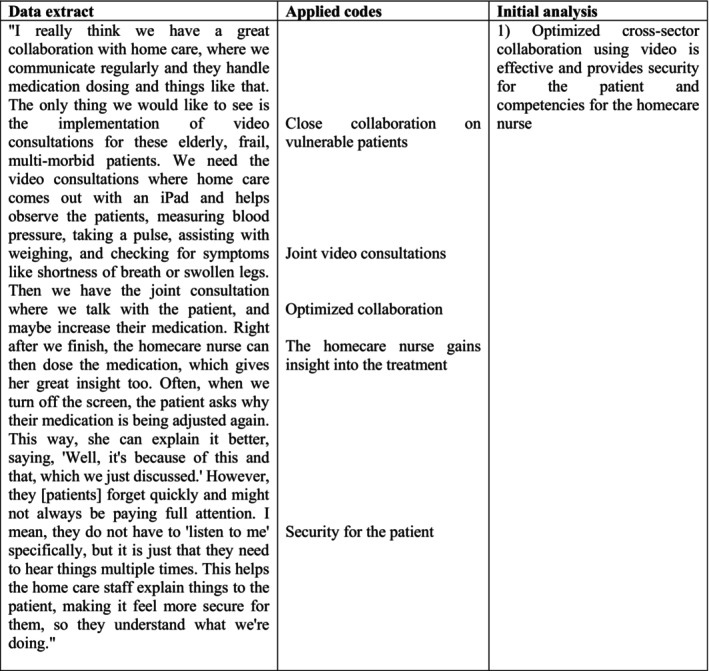
From data extract to initial analysis—Phases 2 and 3.

All interviews were transcribed verbatim either by N.K.J. or by the clinical specialist in nursing who also conducted the interviews. The initial phase was performed by N.K.J., engaging deeply with all of the dataset to achieve a comprehensive understanding and identifying meanings and patterns. N.K.J. proceeded with the second phase using an inductive, data‐driven approach (Braun and Clarke 2022) with NVIVO software applied for systematic data organization and manual coding (Jackson and Bazeley 2019). Figure 1 illustrates an example of the process. Phases 3 and 4 were carried out in close collaboration with A.D.S., systematically examining codes and patterns from the second phase to identify and consolidate potential themes. Relevant data for each theme were gathered, and potential themes and sub‐themes were critically discussed. In Phase 5, M.J.R. was involved in collaboratively refining and reaching consensus on the final themes and sub‐themes. During Phases 3–5, thematic maps were used to structure and visualize the development of themes. To enhance transparency, visual representations of the thematic development are provided as Supporting Information (see “Thematic maps” in the Supporting Information). Finally, Phase 6 involved the detailed composition of the analytical report.

### 2.6 Reflexivity

The research team consisted of a PhD student (N.K.J.), associate professors (M.J.R., A.D.S. and S.A.) and a professor (K.E.), with expertise in chronic illness, telemedicine, and/or qualitative research. Their professional backgrounds in nursing or medicine provided valuable context but also influenced data interpretation. To enhance reflexivity, the three researchers involved in the analysis engaged in ongoing discussions to examine how their professional positions and perspectives influenced theme development. The other authors contributed to analytical discussions, ensuring coherence and relevance.

### 2.7 Ethical Considerations

The study was registered with the Danish Data Protection Agency (reference number: 23/7217). In accordance with Danish regulations, a formal inquiry was submitted to the Regional Scientific Ethical Committee for Southern Denmark, which confirmed that the study did not require ethical approval. Participation in the project was voluntary and performed according to the Helsinki Declaration (Association 2024). Participants signed an informed consent declaration, and they had the opportunity to withdraw their consent at any time without any consequences or needing to provide an explanation. To ensure confidentiality, all interviews were pseudonymized during transcription, with personally identifying information removed before analysis. Only the interviewer and first author had access to identifiable data; other team members worked with pseudonymized data. All data was stored securely in Open Patient data Explorative Network (OPEN) and SharePoint, with access limited to the research team.

## 3 Results

A total of 12 HCPs were invited and agreed to participate in this study. Of the participants, nine were women, and the mean professional seniority for all participants was 29 years (ranging from 5 to 48 years). The participants consisted of eight nurses and four physicians, with half of them currently holding leadership positions. Nine of the participants were employed in a hospital setting, while the remaining three worked in the primary care sector. In the Danish healthcare system, municipal services are part of the primary care sector and include responsibilities such as home nursing, rehabilitation, and preventive care. Twelve semi‐structured interviews were conducted, with durations ranging from 26 to 42 min.

Three overall themes were developed: (1) balancing challenges and benefits of virtual care, (2) strengthening cross‐sectoral collaboration, and (3) empowering the patients through telemedicine. The themes related to the HCPs experiences with the use of telemedicine in specialized care and treatment of patients suffering from chronic, symptomatic diseases.

### 3.1 Balancing Challenges and Benefits of Virtual Care

Participants emphasized that the integration of video consultations in outpatient clinics requires new skills to build confidence, particularly in the development of safe relationships and interpretation of non‐verbal cues. These skills were initially seen as challenging, but they were ultimately recognized as valuable competencies:

You really learn a lot from communicating over the screen. You've got to use all your senses, and you really … like … have to be aware of your non‐verbal cues. You're listening to the patient's voice. You're actually using yourself a lot, maybe even more than when you're physically across from a patient. You really need to extend your awareness even more. (HCP 010, hospital)

Different types of communication skills were described as necessary for video consultations compared to in‐person consultations; requiring intensified awareness of body language and attentiveness to the tone of voice. Participants also highlighted that video consultations enable relatives to join discussions, providing valuable support for patients:

Video is really a good way for the relatives to be part of the conversation, too. It makes it much easier for the patient to get the support they need. (HCP 005, hospital)

Relatives were recognized as essential, offering insights, encouragement, and helping to ensure understanding and adherence to treatment plans.

Despite positive experiences, some participants noted that the slow adoption of telemedicine may stem from HCP reluctance. While telephone consultations were often preferred due to familiarity, video consultations were seen as more challenging:

Well, I'd definitely prefer video. I find phone calls a bit challenging, and I can't help but think that there are some barriers. I mean, no one really has a problem booking a phone consultation … we just schedule it without asking the patients the same way … There are courses on how to conduct a video consultation, and that's really great. I've never heard of a course for phone consultations, and it's actually quite tough. (HCP 007, hospital)

Participants described telephone consultations as being used by default without considering patient consent or preference. Meanwhile, HCPs often worried about whether patients could manage video consultations:

As soon as it's about video, I think we all get a bit nervous and wonder, “Oh no, can the patients figure it out?” (HCP 007, hospital)

Interestingly, some participants observed that patients, even older ones, were often more open to telemedicine than assumed:

The past few years have shown me that the barrier is often with the healthcare professionals, not the patients. We even have people over 80 navigating health platforms … So, I think we should just go for it. (HCP 004, hospital)

Participants emphasized that the greatest barrier might be the assumptions of HCPs about patients being unwilling or unable to use telemedicine, but that it remained crucial to offer the option. If patients struggled to manage on their own, collaboration with the primary care sector could be a viable solution.

Some participants with long‐term experience expressed confidence and familiarity with using telemedicine. However, other participants without prior experience questioned the efficacy and data quality but acknowledged the potential:

It's hard to say if video would give more benefit than just a phone call … maybe it could, you know? Like, if you've got a telemedicine setup where you can also get some data, then it might help with decision support. Like, if you're unsure during a consultation, you could check their oxygen levels. You can't do that over the phone. (HCP 011, hospital)

Participants recognized the value in combining home monitoring with video consultations because it allowed patients to measure data at home, which improved decision‐making during consultations.

Challenges with telemedicine were highlighted for elderly or vulnerable patients, often due to technical barriers. A potential solution could be close collaboration with primary care, as telemedicine is especially crucial for this group to prevent unnecessary hospital visits. While participants viewed telemedicine positively for these patients, they raised concerns about over‐reliance. Primary care participants emphasized balancing telemedicine with in‐person visits to avoid missing critical observations:

There will be things I don't see [on video] that I might notice if I visit them at home … And on days when they're not doing well, they might say, “Oh, I can't get a signal or make the video work.” There are some things that might be harder for us to catch early on. (HCP 002, municipality)

Participants expressed worries that overusing telemedicine could leave chronically ill patients isolated and potentially increase loneliness. Participants stressed the importance of understanding the perspective of the patient, particularly their sense of security with using telemedicine:

What I'd be curious about is how people actually feel about it, if they feel it's just as safe as having a healthcare professional come out to see them … It's all about feeling safe. Do they feel that same safety talking to someone through a screen? It's really their experience that matters. (HCP 002, municipality)

Understanding the patient experience should guide telemedicine practice. Yet, uncertainty remained on whether the patient feels as safe during video consultations as in‐person consultations.

### 3.2 Strengthening Cross‐Sectoral Collaboration

Participants highlighted the importance of regular cross‐sectoral meetings to strengthen collaboration in the care for patients with chronic diseases. While physical meetings were seen as most effective, they acknowledged that video meetings were often more practical:

It's out in the municipalities where we have the close partners … well, we only write to each other there, and we don't talk the same way as we do in our daily work and collaboration here [at the hospital]. (HCP 008, hospital)

Written communication alone made it difficult to build relationships or collaborate effectively, and tight schedules and varying working hours between sectors further complicated the coordination. Participants emphasized the need to break down sector boundaries to create a seamless patient experience:

I think we need to break down all these sector boundaries and have much closer collaboration, so the patient doesn't feel like they're going to different places, but that it's one continuous process (HCP 005, hospital)

Participants viewed video consultations and virtual meetings as potential tools to support more continuous collaboration between sectors. A more cohesive patient journey was needed without constraints from different sectors. A way to promote continuity between sectors might be through a shared electronic patient record. However, in the absence of such systems, establishing clear collaboration agreements and defining areas of responsibility are essential. A participant from the primary care sector described how their competencies were sometimes underestimated:

I don't always know if the hospital knows what competencies we have [in the municipality], sometimes it feels like they don't think we can figure things out. And the whole thing with having joint video meetings before the patient is discharged, that can be good for some relationships, but at the end of the day, we have competencies here that we can totally handle, as long as we get the right information. (HCP 002, municipality)

Video meetings before discharge were described as a practical solution that could improve information flow and mutual understanding between sectors. To use their competencies effectively, primary care nurses needed accurate information, which could be facilitated through joint video meetings with shared agendas prior to discharge. This could improve the cross‐sectoral collaboration. A lack of understanding of the workflow for each sector also created challenges, as tasks that were simple in one sector could be complex in another:

We often experience that they [patients] come home without a plan … Maybe the wrong medication has been sent home with them, and we understand the hospital is busy. But if we're going to get better at ensuring continuity between primary and secondary care, something's got to change. (HCP 002, municipality)

Errors at discharge, such as incorrect medication or incomplete treatment plans, often disrupted continuity and created challenges for primary care. Video meetings were described as a way to mitigate these challenges by enabling timely clarification and shared planning. This further underscores the need for improved planning and communication to ensure a seamless patient pathway.

### 3.3 Empowering the Patients Through Telemedicine

Self‐management plans were found to be effective tools for empowering patients to understand and manage their condition. Participants emphasized that teaching patients to recognize symptoms and respond appropriately ensured that they could handle potential deterioration. This required active patient engagement, such as measuring blood pressure, saturation, and weight before video consultations.

The courses we have require that they [patients] have done their homework, and a downside can be that sometimes they forget it, and then you don't get the data you need from the patient. (HCP 008, hospital)

When patients provided accurate data, video consultations combined with home monitoring enabled early detection of deteriorations, allowing treatment at home:

If it can help detect exacerbations before they require hospitalization … it's better for the patient. Treating them earlier at home is a huge benefit. (HCP 011, hospital)

While home monitoring offered advantages, participants expressed concerns about the accuracy of measurements, which could lead to uncertainty for both patients and HCPs. For example, incorrect oxygen saturation readings due to factors like cold fingers could cause unnecessary anxiety:

If you take too many saturations without the patient being completely at rest, you might end up seeing a saturation level that's too low, which can cause anxiety for both the patient and the HCP. (HCP 011, hospital)

Inaccurate measurements and the lack of direct clinician assessment posed challenges. However, continuous monitoring was found to be beneficial, allowing patients to upload real‐time data instead of recalling symptoms from memory:

I'd really like to get more data directly from the patients … It would be much easier than asking them how they've been over the past 14 days. (HCP 004, hospital)

Participants described video consultations accompanied by home monitoring as an optimal solution between phone consultations and in‐person visits. This approach reduced the need for frequent hospital visits but maintained patient‐centered care.

## 4 Discussion

This study explored HCPs perspectives on video consultations and home monitoring in the care of CHF and COPD patients. Video consultations were generally viewed positively but required confidence and skills for effective use. In contrast, digital home monitoring faced more skepticism, particularly regarding the reliability of patient‐measured data. However, home monitoring also provided some benefits as it enhanced patient self‐care and improved decision‐making during consultations. Our findings suggest that video consultations and home monitoring can complement each other, enhancing communication and enabling clinical decision‐making. Cross‐sectoral collaboration, such as shared virtual meeting spaces, was valuable for improving care coordination. However, cultural gaps between hospitals and primary care, along with differing perceptions of roles, remain key challenges. Our findings also highlight the need for self‐management plans with reliable measurements to empower patients and prevent hospitalizations. Despite the extensive knowledge of digital solutions, significant challenges persist in integrating these tools into clinical practice. Several studies have explored the patient perspective, highlighting that many patients consider reduced hospital visits a key advantage of video consultations (Assing Hvidt et al. 2023; Schultz et al. 2024; Aamodt, Strömberg, et al. 2020). Despite the patient‐perceived benefits, implementing digital solutions faces considerable barriers. Therefore, the perspectives of HCPs need to be explored, especially in the context of rapid advancements in digital health technologies.

Recent data from the Danish Agency for Digitalization indicates that 89% of individuals aged 16–89 use smartphones to access the internet, reflecting a high level of digital familiarity within the population (Digitalisation 2024). Previous research has identified various barriers to the adoption of digital tools in healthcare. Studies on the attitudes of general practitioners toward video consultations have revealed reluctance among some HCPs to adopt new technologies (Assing Hvidt et al. 2023; Björndell and Premberg 2021; Donaghy et al. 2019; Nguyen et al. 2024). This aligns with our findings, where HCPs expressed doubts about whether patients could handle technology effectively and deliver accurate, reliable measurements. Similarly, a recent study identified barriers among HCPs regarding video consultations, even when patients preferred such modalities (Schultz et al. 2024). Our findings support these results and demonstrate that resistance often stems from the HCPs themselves. These concerns may relate to fears of appearing incompetent or preconceived notions that video consultations are an inferior option for the patients (Assing Hvidt et al. 2023). Research highlights that such assumptions may reflect implicit biases among HCPs. An American survey suggested that HCPs sometimes avoid offering video consultations to patients they assume may struggle with the technology, inadvertently limiting the options (Ganguli et al. 2023). These findings underscore the need for more inclusive practices where patients are given the opportunity to choose the modality that suits their preferences. A recent review on nurse experiences with video consultations and home monitoring in patients with CHF reaffirmed these challenges. It highlighted how virtual consultations can disrupt traditional professional roles and affect their ability to build and maintain strong patient relationships (Rosenstrøm et al. 2024). It emphasized the need for continuous development of competencies to overcome these challenges, consistent with our findings. To address these challenges, practical strategies are essential. Research shows that targeted staff training in role adaptation and digital competencies can enhance confidence and collaboration. Involving HCPs in co‐design ensures telemedicine solutions are user‐friendly and clinically relevant (Brewster et al. 2014). Effective leadership, system integration, and technical support are vital for adoption. In countries with lower digital health maturity or different healthcare structures, these factors are even more critical, with adoption hindered by limited internet access, infrastructure, and digital literacy. Locally adapted solutions and political prioritization are necessary (Masterson Creber et al. 2023).

A participant in our study emphasized that HCPs in general did not express significant concerns about missing critical information during telephone consultations or mistrusting the self‐reported measurements, a finding consistent with previous research (Nguyen et al. 2024). However, in their reflections on video consultations and digital home monitoring, participants expressed notable concerns about data accuracy. Receiving reliable data were considered essential for successful digital home monitoring, as inaccuracies could lead to uncertainty for both patients and HCPs. These emerging concerns about invalid data from digital home monitoring may arise from a lack of familiarity with the new technology. Interestingly, participants suggested that combining video consultations with digital home monitoring might address some of these concerns, as this approach enables HCPs to integrate clinical judgment with patient‐provided data. This combination could provide a more comprehensive understanding of the patient condition compared with telephone consultations alone. Similarly, another study highlighted that the ability to see each other on screen was perceived positively, as it enabled HCPs to identify changes over time (Funderskov et al. 2019).

The growing demand for virtual consultations is driven by HCP shortages and an aging population with an increasing prevalence of chronic diseases. Tailored interventions are urgently needed to enhance readiness among HCPs, while strengthening cross‐sectoral collaboration is equally critical to ensuring a robust and reliable healthcare system. Our study highlights the potential of digital solutions to improve collaboration in managing patients with chronic conditions; an example could be through further development of self‐management plans, which have been shown in a previous study to enhance patient empowerment (Aamodt, Strömberg, et al. 2020). Similarly, a study on older patients with CHF found that home monitoring combined with proper guidance could enhance confidence in recognizing symptoms of deterioration (Jaana et al. 2019). Video consultations have also been found to facilitate closer collaboration across sectors, enabling more effective sharing of responsibilities, particularly appreciated by nurses in the primary care sector (Funderskov et al. 2019). Our findings support these observations, highlighting that video meetings can strengthen cross‐sectoral collaboration by overcoming communication barriers and improving continuity of care. Petersen et al. further noted their value in discharge planning, where real‐time dialogue between sectors ensures patient safety and mutual understanding (Petersen et al. 2019).

While cultural differences across sectors are expected, our findings reveal underlying hierarchical disparities, with some HCPs feeling undervalued by counterparts. Addressing this is crucial. Telemedicine can support collaboration by fostering dialogue and mutual respect. Cross‐sectoral visits may enhance understanding of roles and work cultures (Petersen et al. 2019). Organizational champions can further strengthen collaboration by bridging communication gaps and driving system change (Hendy and Barlow 2012). Recognizing differences in care values is also key to aligning practices and ensuring continuity (Petersen et al. 2019).

### 4.1 Strengths and Limitations

A key strength of this study was the inclusion of nurses, physicians, and leaders from both hospital and municipal settings, offering diverse insights into cross‐sectoral collaboration and telemedicine. This diversity enabled a deeper understanding of professional roles and organizational dynamics. Leader perspectives were particularly valuable for understanding implementation processes. The collaborative data analysis by three researchers further enhanced analytical rigor and reduced the risk of bias.

The participant composition, with 50% in leadership roles, was both a strength and a limitation. While it provided strategic and managerial insights, it may have underrepresented frontline experiences and everyday clinical challenges. Several leaders held dual roles, offering both clinical and managerial perspectives. Additional limitations include the single‐site setting, which supports in‐depth analysis but limits transferability. Furthermore, the low number of physicians may have limited the representation of medical perspectives on video consultations and cross‐sectoral collaboration. The overrepresentation of nurses and the limited number of physicians may have skewed findings toward nursing perspectives, though this reflects current practice, where nurses often lead telemedicine use. Only three participants were from the municipality, possibly limiting primary care perspectives; however, their relevant experience makes them representative of the local context. Finally, the interviewer's professional relationship with three participants may have introduced bias, though this was mitigated by the collaborative and reflexive analytic process.

## 5 Conclusion

This study emphasizes the perspectives of HCPs on the use of video consultations and digital home monitoring in the chronic care of patients with CHF and COPD. Unfamiliarity with new technologies can foster concerns, mistrust, and barriers to adoption in clinical practice. Addressing these challenges requires targeted education and hands‐on training to build confidence and competence among HCPs for integrating telemedicine effectively into their workflows. Although cross‐sectoral collaboration in healthcare is complex, it is essential for ensuring efficient patient journeys. This underscores the need for mutual understanding and greater awareness of the competencies from each sector. Bridging these gaps can strengthen cross‐sectoral relationships, build trust, and ultimately improve patient outcomes. Future research should provide deeper insights and expand data to guide strategies for integrating telemedicine into clinical and cross‐sectoral settings.

## Relevance for Clinical Practice

This study highlights the significant benefits of telemedicine, while addressing the complex challenges of implementation. A key issue is the need for comprehensive skills training to build confidence among HCPs and maintain high standards of professionalism. Additionally, cultural differences in cross‐sectoral collaboration pose barriers to establishing seamless digital patient pathways. To address these barriers, targeted role‐specific training, early involvement of clinical leaders and frontline staff, and the appointment of organizational champions may enhance communication and continuity across sectors (Brewster et al. 2014; Hendy and Barlow 2012; Petersen et al. 2019).

## Author Contributions

Conceptualization: N.K.J., M.J.R., A.D.S., and K.E. Data curation: N.K.J. Formal analysis: N.K.J., M.J.R., and A.D.S. Funding acquisition: N.K.J. and A.D.S. Investigation: N.K.J. Methodology: N.K.J., M.J.R., and A.D.S. Project administration: N.K.J. Supervision: M.J.R., A.D.S., S.A., and K.E. Validation: M.J.R. and A.D.S. Visualization: N.K.J. and K.S.O. Writing – original draft: N.K.J. Writing – review and editing: M.J.R., A.D.S., K.S.O., S.A., and K.E. All authors have read and approved the final version of the manuscript.

## Conflicts of Interest

The authors declare no conflicts of interest.

## Supporting information


Data S1.

## Data Availability

Due to the sensitive and potentially identifiable nature of the qualitative interview data, and because participants did not provide explicit consent for data sharing beyond the research team, the data are not publicly available.

## Semi‐Structured Interview Guide

### 1

**Table:**

Topic	Questions	Probes
Experience with using telemedicine	How satisfied are you in general with telemedicine solutions: video consultations and home monitoring?	
	How confident do you feel using telemedicine?	
	How willing are you to use telemedicine? How would you describe your current willingness to use it in the future?	Explore reasons for changes in willingness
	What did you think about the information available to you via telemedicine?	
Effects of telemedicine on patients, care and care processes	How do you currently use telemedicine solutions in your work? How has it affected your work?	
	What are the main benefits of telemedicine for patients with advanced chronic conditions?	
	How has home monitoring influenced your patients' attitudes toward their illness?	For example: Has it helped them understand the illness or take more control of their health? Examples?
	What are the benefits of using telemedicine for your own work and patient care?	
	What are the main barriers or challenges with telemedicine?	For HCPs, patients, relatives?
	How has telemedicine affected communication and coordination with other healthcare professionals?	Any improvements? Examples?
	How has telemedicine influenced the way you communicate with patients?	Any changes? Examples?
	How has it affected task distribution and care responsibilities, particularly for nurses?	Any changes?
	How has it supported integrated care for this patient group?	
	To what extent has telemedicine increased patient involvement in decision‐making?	
Implementation, future perspectives, and adoption	From your perspective, what are the main benefits for future healthcare providers in using telemedicine?	
	What is needed to increase usability and acceptance of telemedicine among healthcare professionals in the future?	
Debriefing	We are now finishing the interview. Do you have any additional thoughts about the topics we've discussed that you haven't mentioned yet, but would like to share?	Thank you for your participation
