# Supplementary figures and images for: Healthcare Professionals Perspectives on Telemedicine for Patients With Chronic Diseases: A Qualitative Study
Source: Nurs Health Sci. 2025 Jun 4;27(2):e70157. doi: 10.1111/nhs.70157 (PMC12138158; doi:10.1111/nhs.70157)

**Supplementary Material
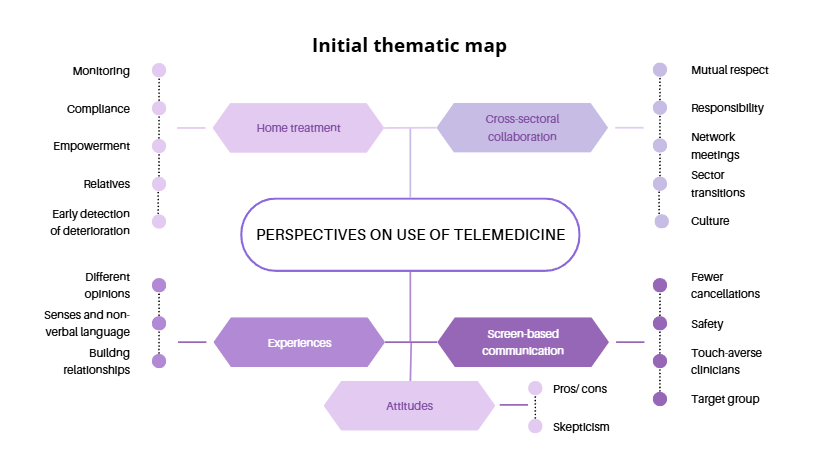
: *Thematic maps***


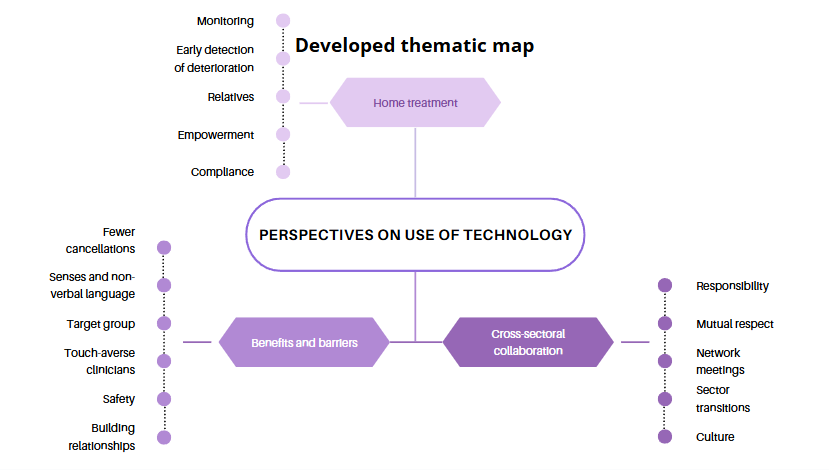


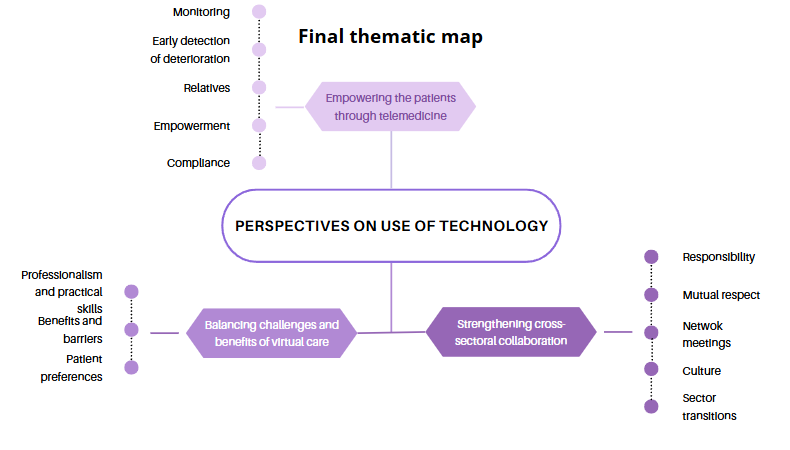

Supplement: Supplementary file 1 — Data S1. [file NHS-27-e70157-s001.docx]
